# Addressing the underdiagnosis of familial hypercholesterolemia: A mixed methods study exploring the knowledge and practice behaviors of cardiology healthcare providers

**DOI:** 10.1017/cts.2023.519

**Published:** 2023-04-05

**Authors:** Isha Kalia, Ronald Shope, Muredach Reilly, Lisa Schwartz

**Affiliations:** 1 Columbia University Irving Medical Center, New York, NY, USA; 2 George Washington University, Washington, DC, USA

**Keywords:** Familial hypercholesterolemia, knowledge, practice behaviors, cardiology, theory of planned behavior, knowledge to action, cardiology healthcare providers

## Abstract

**Background:**

Familial hypercholesterolemia (FH) is vastly underdiagnosed and causes an increased risk for sudden cardiac death. Cardiology providers (CHCPs) are in an ideal position to care for FH patients. This research aimed to understand the practice behaviors of CHCPs in the screening, diagnosis, and management of FH.

**Methods:**

Adaptation of an existing FH knowledge tool guided survey development. The results of the quantitative survey guided development of the interview protocol. CHCPs were recruited in the Division of Cardiology at Columbia University Irving Medical Center (CUIMC). A review of the educational materials offered by the Division of Cardiology was conducted to identify FH knowledge domains presented.

**Results:**

CHCPs with MDs, at CUIMC for 6–10 years, in clinical practice for 1–5 years, and in inpatient services had the highest average total knowledge scores. CHCPs with RNs, at CUIMC for less than 1 year, in clinical practice for 6–10 years, and in Cath Lab had the lowest average knowledge scores. Four themes emerged – variability in FH care; issues related to addressing FH at institutional, practice setting, and individual levels; importance of identifying FH early; and intervention approaches to overcome barriers to caring for FH patients in cardiology. CHCPs with MDs or with experiential FH knowledge described FH care beyond screening. The document review revealed that only MDs were provided lectures pertaining to FH.

**Conclusions:**

Future interventions should increase didactic and experiential FH knowledge incorporating institutional, local, and national FH resources. Improving FH care can reduce FH-related morbidity and mortality, as well as improve FH health outcomes.

## Background

Familial hypercholesterolemia (FH) is a genetic disorder that causes lifelong elevated low-density lipoprotein cholesterol (LDL-C) [1–6]. This chronic hypercholesterolemia causes individuals with FH to have an increased risk for coronary heart disease, myocardial infarctions, and sudden cardiac death [3,7]. Early-onset treatment and management of FH have been shown to reduce the rates of adverse cardiovascular events to that of the general population [1,4,8]. Thus, it is essential to initiate early intervention to reduce acute and chronic complications associated with FH.

While FH is common within the general population with a prevalence of 1 in 200 to 1 in 250, it is a vastly underdiagnosed and undertreated condition [2,8–14]. Barriers to diagnosis include limited provider knowledge of FH, lack of consensus among diagnostic criteria, underutilization of genetic testing, and time constraints in clinical encounters [6,10,11,14–17].

Given the concurrence of underdiagnosis and adverse cardiovascular outcomes associated with FH, cardiology healthcare providers (CHCPs) in the United States are in an ideal position to not only screen cardiology patients for FH but also to diagnose and manage FH patients [15]. While the prevalence of FH within the general population is estimated at 1 in 250, research has shown the pooled prevalence among those with atherosclerotic cardiovascular disease to be 18 times higher than the general population with a prevalence of 1 in 17 [18].

Even though multiple diagnostic criteria exist for FH, and an institutional electronic health record system is in place, the Division of Cardiology at Columbia University Irving Medical Center (CUIMC) does not have a systematic way to screen, diagnose, or manage patients with FH. Developing a systematic way to screen and identify FH patients can assist CHCPs in diagnosing and managing FH and can contribute to early intervention strategies that can mitigate FH-associated adverse health outcomes.

This mixed methods research study aimed to understand current knowledge and practice behaviors among CHCPs in the screening, diagnosis, and management of FH. This study was the first comprehensive mixed methods study to explore the knowledge and practice behaviors in addressing FH, as well as potential interventions towards the systematic screening of hypercholesterolemia and diagnosis of FH cases, among CHCPs within a cardiology clinical practice. Ultimately, knowledge gained from this study can inform the implementation of interventions in cardiology practice to assist with systematic screening, diagnosis, and management of FH patients.

## Methods

### Participants

The study population for this research included CHCPs within the Division of Cardiology at CUIMC in Washington Heights, New York. Providers included physicians (Doctor of Medicine, MD; Doctor of Osteopathic Medicine, DO), physician assistants (PA), and nurses (nurse practitioner, NP; registered nurse, RN; licensed practical nurse, LPN) who provided clinical care to patients in the Division of Cardiology at CUIMC.

### Quantitative Survey

Bell *et al*. [19] utilized the Theory of Planned Behavior (TPB) and Knowledge to Action (KTA) frameworks to develop a 19-item tool to assess the knowledge, awareness, and practice of FH among general practitioners in Australia. Of the 19 items, seven items measured knowledge, three items measured awareness, and nine items measured practice [19]. This tool underwent content validation, adaptation, and face validation [20,21]. The adapted and validated FH tool included 11-knowledge items, five awareness items, and nine practice items, consisting of a total of 25 items [20,21]. The Kuder Richardson formula-20 internal consistency coefficient for the overall instrument was 0.79, including the following subdomains: knowledge (0.53), awareness (0.76), and practice (0.61) [20,21]. With regards to test-retest reliability, Cohen’s kappa coefficient for the instrument was 0.76, with subdomain measurements including 0.82 (knowledge), 0.81 (awareness), and 0.76 (practice) [20,21].

For the purposes of this study, the survey included an adaptation of the Azraii tool. Awareness items in the tool included familiarity with FH, FH guidelines, and diagnostic criteria [20,21]. However, these items did not align with the attitudes construct within the TPB, which includes “the degree to which a person has a favorable or unfavorable evaluation or appraisal of the behavior in question” [22]. FH practice behaviors were further explored during the qualitative interviews. Therefore, items measuring awareness and practice in the Azraii tool were removed. The final survey for this study included only the remaining 11-knowledge items from the Azraii tool with a total possible score of 19 with a point per correct response including multiple correct responses per question [22]. The second round of adaptation included changing lipid profile measurements as well as modifying practice guidelines and diagnostic criteria to the United States context.

### Qualitative Semi-Structured Interview Protocol

Since the aim of this phase of research was to elucidate the factors that influence FH screening, diagnosis, and management at CUIMC, a qualitative case study approach using a constructivist paradigm offered opportunities for a more complete, nuanced, contextual, and detailed understanding of CHCPs’ perceptions [23,24]. Final design of the interview guide was informed by the results of the quantitative survey, expert review, and based on the conceptual frameworks (KTA and TPB) for this research [11,22,26,27].

### Sampling and Recruitment

Convenience sampling through CUIMC’s Division of Cardiology email listserv was utilized for the quantitative phase of this study [23,24]. The listserv included eligible participants (134 MD/DO, 57 NP/RN, and 41 PA) within 15 subdivisions in the Division of Cardiology. To increase response rate, snowball sampling was employed by asking respondents to the survey who expressed willingness to participate in the qualitative interview to forward the survey to their colleagues at CUIMC.

The unit of analysis was the CHCP (MD, DO, PA, NP, LPN, RN), and each case was bound by healthcare setting (CUIMC) and specialty (cardiology). To obtain a diversity of cases, increase the richness of the data, and aim for maximum variation, a criterion sampling approach was utilized [23,24,28]. To allow for maximum variation sampling, a subsample of 20 participants from the quantitative survey was created using criteria including total knowledge score, professional degree/license, and subdivision. Snowball sampling was used to recruit participants for the qualitative interviews through professional connections at CUIMC, as well as through participants who agreed to participate in the second phase of the research study [23,24].

### Study Procedure

All research activities were approved by the CUIMC and George Washington University Institutional Review Boards (IRBs; IRB-AAAU0047). A mixed methods, sequential, explanatory case study design was conducted. Within the quantitative phase, a validated FH knowledge survey was distributed to healthcare providers across the Division of Cardiology at CUIMC. Results from the quantitative survey and the conceptual frameworks (KTA and TPB) informed the development of interview questions used in the qualitative phase of this study. The qualitative arm included semi-structured interviews with individual CHCPs who participated in the quantitative phase and agreed to be contacted for the second phase of the study. Quantitative survey data, qualitative interview data, and other data sources including a review of current protocols, training documents, and educational materials provided by the Division of Cardiology were compared within the integrative phase of this study.

### Data Analytic Plan

Quantitative data from the survey instrument were analyzed using descriptive statistics. Frequency counts of provider type, subdivision of cardiology, years in clinical practice, and years in clinical practice at CUIMC were calculated. Survey responses for the 11-knowledge items were added to produce a total knowledge score for each participant. A total sum knowledge score of up to 19 was calculated for each completed survey for use in data analysis. The range of knowledge scores for all participants was reviewed to identify high and low thresholds and support variation among interview participants. The two-eligibility, 11-knowledge and four-demographic, items were marked as mandatory for participants to complete in Qualtrics. Incomplete surveys were removed from data analysis.

Interview transcripts were single-coded with Dedoose [29] software using an *a priori* coding schema that was informed by the TPB constructs [22–24]. In addition to interview transcripts, other data sources included a document review of current protocols, training documents, and educational materials provided by the Division of Cardiology from 2018 to 2022. Leadership of each participant subgroup (i.e., attendings, fellows, nurses) was contacted to determine what resources (i.e., webinars, grand rounds, training documents) were provided to members within the division. These resources were reviewed and analyzed descriptively to determine if FH content was included, and if so, what specific content areas pertaining to FH (i.e., diagnostic criteria, management options) were discussed.

## Results

### Quantitative Survey

A total of 232 eligible participants (134 MD/DO, 57 NP/RN, and 41 PA) received an introductory study email including the Qualtrics link to recruit participants into the quantitative phase. Seventy-nine (79) respondents completed the inclusion/exclusion criteria. Nine (9) had incomplete responses that could not be analyzed, resulting in 70 completed survey responses that were available for analysis, corresponding to a response rate of 30.2%.

### Total Knowledge Scores

The survey consisted of 11-knowledge items, with one point given per each correct response; some items included multiple correct responses [20,21]. Survey responses for the 11-knowledge items were added to produce a total knowledge score of up to 19 possible for each participant. CHCPs with MDs (*x̄* = 12.5), at CUIMC for 6–10 years (*x̄* = 11.7), in clinical practice for 1–5 years (*x̄* = 11.4), and within the subdivision of Inpatient Services (*x̄* = 15.5) had the highest average total knowledge scores. CHCPs with a professional degree or license of RN (*x̄* = 7.5), at CUIMC for less than 1 year (*x̄* = 9.4), in clinical practice for 6–10 years (*x̄* = 9.8), and within the subdivision of Cath Lab (*x̄* = 8.7) had the lowest average knowledge scores.

### Interview Participant Recruitment

Twenty-four CHCPs who completed the quantitative survey expressed interest in participating in follow-up individual semi-structured interviews. A subsample consisting of twenty-one participants was selected to confirm maximum variation among study participants. Twenty-one participants were contacted via email to schedule the qualitative interview via secure Zoom audio conferencing [30]. Twenty CHCPs responded, scheduled, and completed an interview corresponding to a 95.2% response rate.

A total sum knowledge score was calculated for each completed survey. Surveys with a total knowledge score of zero to nine, receiving a score of 50.0% or less, were categorized as low knowledge. Surveys with a total knowledge score from 10 to 19, receiving a score of greater than 50.0%, were categorized as high knowledge. Interview participants were equally distributed by total knowledge score (i.e., 10 interviews were conducted with CHCPs with high knowledge scores and 10 interviews were conducted with CHCPs with low knowledge scores).

### Thematic Analysis

Using both deductive and inductive coding, four overarching themes related to the practice behaviors of CHCPs in the screening, diagnosis, or management of FH in cardiology clinical practice were identified.

**Theme 1: Variability in FH Care.** The ability to provide comprehensive care to FH patients is highly dependent on the ability of CHCPs to first identify patients at risk for FH. Since FH causes elevated LDL-C levels, the lowest threshold for identification is through a lipid panel. Given the clinical practice setting of cardiology and the use of the lipid panel as a screening tool for cardiovascular disease risk, CHCPs frequently discussed the use of the lipid panel in clinical practice. However, CHCPs acknowledged the lack of standard practices for ordering a lipid panel and using the results.

**Theme 2: Importance of Identifying FH Early.** To better understand how the behavioral beliefs and attitudes of CHCPs could influence the care of FH patients, the importance, advantages, and disadvantages of screening, diagnosing, and managing FH in clinical practice were explored. CHCPs had both positive behavioral beliefs and attitudes toward addressing FH. CHCPs emphasized the importance of addressing FH as it would impact their day-to-day practice and the ability to practice preventive medicine. This concept of prevention was first discussed in the context of secondary prevention or reducing the impact of disease in an already affected patient and second through primary prevention or preventing the onset of disease in unaffected family members of FH patients.

**Theme 3: Institutional, Practice Setting, and Individual Issues to Addressing FH.** Even though CHCPs held positive behavioral beliefs and attitudes towards screening, diagnosing, and managing FH, their practice behaviors varied greatly. To better understand this gap, the normative beliefs, subjective norm, control beliefs, perceived behavioral control, and individual and external issues were further explored. A number of barriers and facilitators for CHCPs in the care of FH patients were identified. These barriers and facilitators were organized by institutional-level, practice setting, and individual-level issues.

With regard to institutional-level barriers, CHCPs described not only a lack of awareness of institutional resources but also unfamiliarity with referral mechanisms. CHCPs with MDs were more aware of institutional resources and referral mechanisms and described how working at a large academic medical center was a facilitator to providing comprehensive care to FH patients.

Across practice settings, CHCPs described the lack of messaging regarding if and how to screen, diagnose, or manage patients with FH. This lack of guidance from leadership negatively influenced participants’ normative beliefs and subjective norm. A second practice setting barrier addressed by CHCPs was the context of the clinical encounter. Participants described how specific clinical practice settings were less amenable to caring for patients with FH. Additionally, CHCPs described how the prioritization of care within a clinical encounter could serve as a barrier to addressing FH in practice. For instance, CHCPs could have higher priority acute problems to address within an inpatient setting as opposed to an outpatient clinical encounter.

While CHCPs described many practice setting barriers to providing care for FH patients, one facilitator that was discussed was addressing FH in practice settings where FH could be the underlying reason for the clinical encounter. Providers discussed how FH care could be targeted to clinical practice settings such as cardiac catheterization, interventional cardiology, or surgery where the adverse effects of hypercholesterolemia are addressed, as opposed to electrophysiology or congenital heart disease in which chronic hypercholesterolemia is not the primary cause of the visit.

In addition to institution-level and practice setting issues, CHCPs also described barriers and facilitators at the individual level. CHCPs highlighted both CHCP and patient-related issues that influenced their ability to provide FH care. With regard to barriers at the patient level, CHCPs described how a patient’s insurance coverage, health literacy level, hesitancy to begin medication and medication compliance affect a provider’s ability to screen, diagnose, or manage FH in clinical practice. Participants also discussed CHCP-specific issues that serve as barriers to providing FH care including feelings around prescribing medications, difficulty of managing complex cases, finding FH-specific information, and their lack of didactic and experiential FH knowledge.

CHCPs also highlighted facilitators at the individual level that influenced a CHCP’s ability to provide FH care. With regard to a patient-level facilitator, CHCPs described how a diagnosis of FH could reduce barriers related to the prior authorization process, insurance coverage, and cost of medications. Another individual-level facilitator that arose from the qualitative interviews was CHCPs’ interpersonal relationships with other CHCPs as well as with pharmaceutical sales representatives. Specifically, CHCPs described how their relationships with other CHCPs address institution, practice, and individual-level barriers such as understanding institutional resources, mechanisms for referral, guidance from leadership, and knowledge of FH. CHCPs also described how their interpersonal relationships with pharmaceutical sales representatives can assist them in addressing barriers to FH care such as insurance coverage, cost of medication, prior authorization processes, and knowledge of FH.

**Theme 4: Overcoming Barriers.** CHCPs described several ways to overcome institutional, practice setting, and individual-level barriers including the use of a referral system, education, and EHR applications. A need for a referral system was described by CHCPs outside of lipidology to provide a seamless process to integrate these patients into a specialty clinic. Additionally, a referral system was described by CHCPs within a lipid specialty who described a need for an increase in patient volume.

Another intervention often described by CHCPs was the use of educational initiatives such as lecture series, continuing medical education events, or case studies to improve the individual barrier of CHCPs’ low FH knowledge. When considering the advantages of educational interventions, CHCPs described how education can overcome individual-level provider barriers such as increasing cognitive awareness and knowledge of FH, as well as provide ease of access to current practice guidelines. CHCPs recognized that a significant disadvantage to these educational initiatives includes planning, organizing, and scheduling these events to maximize participant attendance, as well as the voluntary nature of these activities, and how voluntariness can affect attendance.

Lastly, CHCPs described using EHR technology to assist with the screening, diagnosis, and referral of FH cases. Participants most often discussed using an alert within the EHR system. CHCPs described how this alert could assist in identifying patients based off LDL-C alone, stratifying low and high-risk patients, diagnosing patients using diagnostic criteria such as Dutch Lipid Clinic Network criteria, and managing patients previously diagnosed with FH. Advantages of alerts in the EHR described by CHCPs were that alerts were part of providers’ clinical workflow, increased the cognitive awareness of FH for providers, and prevented providers from deferring FH care based on individual, practice setting, or institutional barriers. While CHCPs recognized that an FH alert could be valuable for improving the care of FH patients, they also addressed the disadvantages of using alerts in the EHR including alert fatigue and not having the baseline knowledge of FH to understand the purpose of the alert.

### Document Review

A review of the educational materials from 2018 to 2022 was conducted to determine the educational resources offered by the Division of Cardiology to CHCPs. These materials were reviewed and analyzed descriptively to determine if FH content was included, and if so, what specific knowledge domains pertaining to FH (i.e., diagnostic criteria, management, prevalence, inheritance) were discussed. The Division of Cardiology at CUIMC offers three main educational opportunities including Cardiology Grand Rounds, Cardiovascular Seminar Series, and a fellowship lecture series.

Cardiology Grand Rounds is a series of lectures offered to a wide variety of CHCPs including MD, DO, PA, NP, LPN, and RNs. Over the course of four years from 2018 to 2022, a total of 60 lectures were provided and none contained content pertaining to FH. Another lecture series offered by the Division of Cardiology is called the Cardiovascular Seminar Series. The audience of this series is CHCPs with professional MD and DO licenses. From 2018 to 2022, a total of 71 lectures were provided and none contained content related to FH.

Finally, the fellowship lecture series was offered to MD and DO CHCPs within the Division of Cardiology. From 2018 to 2022, CHCPs were provided a total of 210 presentations within the fellowship lecture series. Of 210 presentations over the course of four years, four lectures (1.91%) provided educational content related to FH. One FH presentation was given annually from 2018 to 2022. The FH knowledge domains presented within this lecture included a general description of FH, prognosis, prevalence, inheritance, diagnostic criteria, and management options.

## Discussion

**Fig. 1.**
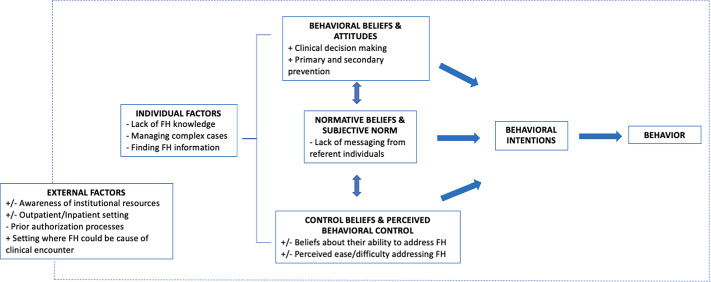
Summary of major findings. FH, familial hypercholesterolemia.

This research was one of the first known studies in the United States that exclusively explored the knowledge and practice behaviors of CHCPs in the screening, diagnosis, and management of patients with FH. While CHCPs expressed the importance of addressing FH in clinical practice, there was a wide range of practice behaviors related to the care of FH patients. Figure 1 highlights the individual, practice setting, and institutional issues identified in this research. Understanding the barriers and facilitators that may influence FH practice behaviors can contribute to the development of future interventions and implementation strategies to help CHCPs address FH in cardiology clinical practice.

One of the most important factors that affected a CHCP’s ability to care for patients with FH highlighted through this research was a provider’s knowledge of FH. As described by participants, there were two ways to obtain FH knowledge: through education (didactic knowledge) or through practice (experiential knowledge). Didactic and experiential exposure could occur in a variety of settings such as in health professional training, clinical rotations, conferences, lecture series, or on the job in clinical practice. Exposure to FH, either through didactic or experiential opportunities, was a critical first step to improve the screening, diagnosis, and management of FH. Without knowledge of the condition, CHCPs will not recognize FH in their clinical encounters, nor will CHCPs be able to screen, diagnose, or manage FH patients.

Beyond CHCPs’ level of FH knowledge, other individual issues that serve as barriers to screening, diagnosing, and managing FH in cardiology practice included providers’ hesitancy about prescribing lipid-lowering medications due to the perceived barriers to get these medications approved, managing complex cardiac cases and prioritization of care, and difficulty finding FH information and resources. Individual-level facilitators for CHCPs were the interpersonal relationships with individuals who had higher levels of FH knowledge such as other CHCPs or pharmaceutical sales representatives. A key underlining aspect of these interpersonal relationships was the necessity for one party to have a high level of FH knowledge, without which these interpersonal relationships may have served as a barrier to FH care.

The lack of FH knowledge expanded beyond FH-specific content such as inheritance, prognosis, and diagnostic criteria and included lack of FH institutional knowledge. Not having knowledge of institutional resources such as lipid clinics or genetic specialists, as well as mechanisms for referrals to these resources, acted as a barrier for CHCPs to provide the standard of care to FH patients. CHCPs described practice setting barriers such as limited time in a clinical encounter and FH being beyond their scope of practice, as barriers to clinical FH care. Therefore, if CHCPs donʼt have the resources (i.e., knowledge, time) to diagnose or manage FH, the minimum that CHCPs could do is refer FH patients to providers with the knowledge, skills, and resources to provide comprehensive FH care.

This research highlighted that having FH content knowledge and FH institutional resource knowledge influences CHCPs’ control beliefs, perceived behavioral control, normative beliefs, and subjective norm. Providers with didactic or experiential FH knowledge had positive control beliefs and higher levels of perceived behavioral control, leading them to provide FH care beyond the point of screening such as making referrals, diagnosing, or managing FH patients. Unfortunately, these CHCPs with high FH content knowledge and institutional resources knowledge were a minority in the qualitative phase of this study. The majority of CHCPs in the qualitative phase, including both low and high knowledge CHCPs, described having a limited knowledge of FH and institutional resources, which negatively influenced their control beliefs and perceived behavioral control, as these CHCPs did not feel equipped with the knowledge, skills, and resources to screen, diagnose, or manage FH patients.

CHCPs also emphasized the lack of messaging from referent individuals such as subdivision leadership, other CHCPs, or Division of Cardiology leadership. This lack of messaging negatively influenced CHCPs’ normative beliefs and subjective norm. Even though participants had positive behavioral beliefs and attitudes toward providing care to FH patients in cardiology practice, the innumerable barriers at the individual, practice setting, and institutional levels negatively influenced CHCPs’ normative beliefs, subjective norm, control beliefs, and perceived behavioral control. While CHCPs expressed the value of addressing FH in clinical practice, their lack of knowledge and resources prevented them from performing behaviors related to screening, diagnosing, and managing FH. This study highlighted that to improve FH patient outcomes at CUIMC, CHCPs not only need to increase their content knowledge of FH but also their knowledge of FH institutional resources.

## Limitations

This research had several limitations. The quantitative phase yielded a total of 70 completed surveys which may not be representative of all subdivisions of Cardiology, cardiology professional degrees/licenses, years of clinical practice, levels of FH knowledge, as well as FH experiences and practice behaviors. Further, selection bias may have occurred among CHCPs who elected to participate in the survey. CHCPs were provided a brief description of the study details in the recruitment email which identified FH as the central topic of study. CHCPs with prior knowledge or experience with FH may have been more likely to participate in the survey. Additionally, the inability to observe participants while taking the survey may have resulted in a miscategorization of CHCPs as high or low knowledge if additional resources such as online reference tools or other CHCPs were utilized to complete the quantitative survey.

Within the qualitative phase, a total of 20 CHCPs participated in semi-structured interviews. Qualitative data provided an in-depth and nuanced understanding of the experiences of CHCPs in the screening, diagnosis, and management of FH. While the sample of 20 individuals represented a diversity of subdivisions, levels of FH knowledge, and professional degrees/licenses, this sample cannot be considered representative of CHCPs broadly. The findings from the quantitative and qualitative phases may not be generalizable to other practice settings (i.e., primary care, pediatrics, family medicine), institutions, or patient populations. However, the use of quantitative and qualitative data provided rich descriptions of cases and allowed readers to determine aspects of this research that could be transferable to other practice settings, institutions, or patient populations. Finally, while this research explored the knowledge and practice behaviors of CHCPs, it neglected to account for the perspective of FH patients. To better understand the factors that affect the screening, diagnosis, and management of FH, the knowledge and health behaviors of FH patients should be further explored.

## Future Research

While research continues to expand our knowledge of the pathology, genetic contributions, and pharmacogenomic treatment options for FH, this study highlighted the necessity for more research addressing T3 chasms of the translational spectrum. If providers on the frontline of patient care are unable to screen or diagnose FH in clinical practice, then how do T1 and T2 research efforts translate to improved health outcomes for patients with FH?

FH patients present to care in a variety of settings. While this study focused on the clinical context of cardiology specifically with CHCPs, future research needs to explore the barriers and facilitators to FH care in other practice settings (i.e., primary care, family medicine, pediatrics), as well as explore the experiences related to screening, diagnosing, and managing FH among other healthcare professionals. Additionally, like many chronic health conditions, diagnosis is simply the first step to improving long-term health outcomes. To minimize the adverse health outcomes associated with FH, future research should explore FH patient experiences to identify patient issues such as the ability to afford medications, health literacy level, and medication compliance that may influence the screening, diagnosis, and management of FH.

This research aimed to elucidate the factors that influence the clinical care of FH in cardiology practice at a single academic medical center. The knowledge gained from this study may be translated to other demographic regions, practice settings, and patient populations. Future research centered on addressing T3 and T4 gaps in FH knowledge can not only bridge translational chasms and inform future research but also make significant progress in addressing the underdiagnosis and undertreatment of FH.
